# Propyl gallate inhibits hepatocellular carcinoma cell growth through the induction of ROS and the activation of autophagy

**DOI:** 10.1371/journal.pone.0210513

**Published:** 2019-01-17

**Authors:** Po-Li Wei, Chien-Yu Huang, Yu-Jia Chang

**Affiliations:** 1 Department of Surgery, College of Medicine, Taipei Medical University, Taipei, Taiwan; 2 Division of Colorectal Surgery, Department of Surgery, Taipei Medical University Hospital, Taipei Medical University, Taipei, Taiwan; 3 Graduate Institute of Cancer Biology and Drug Discovery, Taipei Medical University, Taipei, Taiwan; 4 Cancer Research Center and Translational Laboratory, Department of Medical Research, Taipei Medical University Hospital, Taipei Medical University, Taipei, Taiwan; 5 Division of General Surgery, Department of Surgery, Shuang Ho Hospital, Taipei Medical University, Taipei, Taiwan; 6 Graduate Institute of Clinical Medicine, College of Medicine, Taipei Medical University, Taipei, Taiwan; 7 International PhD Program in Medicine, Taipei Medical University, Taipei, Taiwan; Institute of Biochemistry and Biotechnology, TAIWAN

## Abstract

The poor prognosis of hepatocellular carcinoma (HCC) has been attributed to a high frequency of tumor metastasis and recurrence even after successful surgical resection. With less than 30% of patients benefiting from curative treatment, alternative treatment regimens for patients with advanced HCC are needed. Propyl gallate (PG), a synthetic antioxidant used in preserving food and medicinal preparations, has been shown to induce cancer cell death, but the anticancer effects of PG in HCC are unclear. In the present study, we demonstrated that PG inhibited HCC cell proliferation in vitro and in zebrafish models in vivo in a dose- and time-dependent manner. PG also induced cell apoptosis and increased the number of necrotic cells in a time- and dose-dependent manner as determined using a high-content analysis system. We found that PG also increased the intracellular levels of superoxide and reactive oxidative stress as well as the formation of autophagosomes and lysosomes. Regarding the molecular mechanism, PG did not alter the levels of autophagy-related 5 (ATG5), ATG5/12 or Beclin-1 but increased the rate of the LC3-I to LC3-II conversion, suggesting autophagy induction. PG exposure increased the levels of the pro-apoptotic proteins cleaved caspase-3, cleaved PARP, Bax, and Bad and a decreased level of the anti-apoptotic protein Bcl-2. In conclusion, we demonstrate that PG inhibits HCC cell proliferation through enhanced ROS production and autophagy activation. Finally, PG-treated cells induced cell apoptosis and may be a new candidate for HCC therapy.

## Introduction

Hepatocellular carcinoma (HCC) is the malignant cancer derived from hepatocytes and is the most common cancer worldwide [1]. HCC-related mortality ranks *third* with regard to cancer-related deaths worldwide but ranks second for this statistic in China [2]. Although there are curative treatments, including surgical resection and liver transplantation, less than one third of newly diagnosed patients are candidates for these treatments [3, 4]. Microvascular invasion and occult metastasis after surgical resection lead to the poor outcome of HCC. An alternative treatment for patients with advanced HCC who cannot receive curative treatments, such as surgery, transplantation, transarterial chemoembolization (TACE) or radiofrequency ablation[5], is the multitargeted kinase inhibitor called sorafenib, a drug approved by the Food and Drug Administration (FDA) for advanced HCC. However, sorafenib efficacy is limited by resistance and toxicity [6,7]. Therefore, developing new agents to treat HCC is challenging for researchers [8].

Recent attention has focused on the seeking of safe and effective anti-tumor compounds from Traditional Herb Medicine, and several components isolated from plants possess significant therapeutic efficacy against several cancers [9]. Propyl gallate (PG), propyl-3,4,5-trihydroxybenzoate, a polyphenolic compound family that is synthesized by the condensation of gallic acid and propanol, is commonly used in processed food and cosmetics, hair products, and lubricants (mainly oils and fats) to prevent rancidity and spoilage[10]. PG, similar to superoxide dismutase, shows protective effects against oxidation by hydrogen peroxide and oxygen free radicals via a catalytic effect [11]. Among these effects is the stimulation of oxygen uptake that occurs in electron transport chains on mitochondria and microsome [12]. Previous studies have reported the stimulation of microsomal respiration and inhibition of pyruvate transport, suggesting complex and intense interactions of PG with cellular membranes. PG shows a relatively strong lipophilic character [12–14]. This lipophilicity must confer affinity for organelle membranes, which could also explain the interactions of PG on mitochondria and microsomes [13].

In addition to its antioxidant activity, PG also exhibits various biological abilities, including anti-inflammatory, anti-angiogenic, and anti-tumor effects [15–16]. It is suggested that the cytoprotective / antioxidative functions of PG may change to pro-oxidative, cytotoxic properties in the presence of copper (II) oxide [15–16]. PG induces apoptosis in human leukemia cells [17] and HeLa cells [18] by increasing reactive oxygen species (ROS) levels and/or glutathione (GSH) depletion. The GSH depletion-mediated cell death and ROS production induced by PG in HeLa cells also correlate with the activation of caspases-3/8/9 [19].

PG also plays an important role in autophagy, which serves as a jenus face in cell survival. Autophagy plays an essential role in cellular physiological processes. Under normal cellular homeostasis, autophagy maintains a recycling mechanism at basal rate. Autophagy is stimulated as a stress response to physiological and pathological conditions including hypoxia, inflammation, starvation, and cancer [20, 21]. It is still unclear whether chemotherapy-induced autophagy in tumor cells is a protective response or promotes cell death. First, autophagy acts as a tumor inhibitor through degrading cell components, resulting in second-typed programmed cell death [22]. Second, autophagy functions as a tumor promoter, enhancing tumor cell survival in strict situation[23]. The regulation of autophagy is highly complex, occurring through the Akt/mTOR and MAPK/Erk1/2 signaling pathways [24], and autophagy mediation serves as a potential target for cancer treatment[25].

Our study demonstrates that PG can suppress HCC proliferation through the induction of ROS production in HCC cells after exposure to PG. Additionally, oxidative stress overloading leads to the activation of autophagy and causes cell apoptosis. These findings may provide a new direction for HCC therapy.

## Materials and methods

### Chemicals, reagents, and cell culture

The chemicals used in this study were obtained from Sigma (St. Louis, MO, USA). Antibodies targeting ATG5, Beclin-1, LC3, GAPDH, Bcl-2, and Bax were from Cell Signaling Technology (Danvers, MA, USA), and antibodies targeting cleaved poly (ADP ribose) polymerase (c-PARP) were purchased from Santa Cruz Biotechnology (Santa Cruz, CA, USA). Hep3B and Mahlavu cells were purchased from American Type Culture Collection (ATCC, Manassas, VA, USA), and HepJ5 cells were established by Dr. C. S. Yang as previously described [26]. HCC cell lines (Hep3B, HepJ5, and Mahlavu) were grown in Dulbecco’s modified Eagle’s medium (Life Technologies, Grand Island, NY, USA) supplemented with 10% (v/v) fetal calf serum in a 5% CO_2_ humidified incubator at 37°C.

### Sulforhodamine B (SRB) colorimetric assay for cytotoxicity screening

2×10^4^ cells were seeded in 24-well plates. After overnight incubation, cells treated with of different doses of PG (0–160 μg/ml) or vehicle for 48 h. Next, the cells were fixed with 10% trichloroacetic acid overnight and stained for 30 minutes with protein-bound SRB. The cells were washed twice with 1% acetic acid for removing excess dye. The protein-bound dye was dissolved in 10 mM Tris base solution. Then microplate reader is used for OD measurements at 515 nm (Bio-Rad Laboratories, Hercules, CA, USA).

### Xenotransplantation assay

This assay was performed using the Taiwan Zebrafish Core Facility-Human Disease Model Resource Center. Briefly, at 2 days post-fertilization (dpf), zebrafish embryos were dechorionated and anesthetized with tricaine (0.04 mg/ml; Sigma). HepJ5 or Hep3B cells were harvested and labeled with CFSE fluorescence dye (Vybrant; Invitrogen, Carlsbad, CA, USA). 4.6 nl of tumor cells (approximately 200 cells) were injected into 2 days old zebrafish embryos in the yolk using a Nanoject II Auto-Nanoliter Injector (Drummond Scientific, Broomall, PA, USA). After implantation, the zebrafish embryos were washed with fish water onetime and incubated at 28°C for 1 h. The embryos were then treated with either dH_2_O or PG at doses of 0–40 μg/ml. Fluorescent cells of embryos were checked at 2 h post-implantation and examined at 1 and 3 days post-injection (dpi) by fluorescence microscopy. We use Image J to compare the intensity of 1dpi and 3dpi cells to assess cell proliferation changes percentage by the formula (3 dpi-1dpi)/1 dpi. If the changes are increased more than 5%, we consider the proliferation is increased. If the changes is decreased more than 5%, we consider the proliferation is decreased. If the changes is within + 5%, we consider it is no change.

### The PG effect in HCC was monitored using a fluorescence-based high throughput screening system

Cells (1×10^4^) were seeded into 96-well culture plates (PerkinElmer, Shelton, CT, USA 6005550) overnight. The specific dyes [Hoechst 33258 (f.c. 4 mg/ml), propidium iodide (f.c. 29.9 nM), and NucView 488 (f.c. 5 mM)] were added to monitor the status of live cells and detect viable, necrotic, and apoptotic cells. Different concentrations of PG (0–300 μM) were incubated with HepJ5 cells. Images were captured using ImageXpress at 0, 3, 8, 24, 32, 48 h after treatment and analyzed using the Cell Health module.

### Terminal deoxynucleotidyl transferase-mediated nick end labeling (TUNEL) assay

Cells were plated in six-well plates at 3×10^5^ cells/well overnight and then treated with bromelain or H2O as the vehicle control for 48 h. Cells were harvested and washed with PBS. The cellular DNA fragmentation morphology was detected by a TUNEL assay using an Apo-BrdU in situ DNA Fragmentation Assay Kit (Bio Vision, Mountain View, CA, USA) according to the manufacturer’s instructions. TUNEL-positive cells were then analyzed using fluorescence microscope.

### Total ROS/superoxide detection using the FlexiCyte protocol

ROS were measured using the total ROS/Superoxide Detection Kit (Enzo Life Science, Farmingdale, NY, USA) according to the manufacturer's instructions. Using a combination of two specific fluorescent probes, the kit allows for the real-time observation of global ROS levels, specifically those of superoxide, in living cells. Cells were stained with the two-color ROS Detection Kit and analyzed using the NucleoCounter NC-3000 system (ChemoMetec, Allerod, Denmark). Briefly, 2.4×10^5^ cells were seeded in six-well plates overnight and then treated with 80 μg/ml PG or vehicle. ROS and oxidative stress of the harvested cells were detected by staining with the two fluorescent dyes from the ROS-ID Total ROS/Superoxide detection kit (ENZ-51010; Enzo). In addition, Hoechst 33342 was used to stain the harvested cells to detect the total cell population [27].

### Autophagy detection using an autophagy detection kit

Autophagy was measured using the CYTO-ID Autophagy Detection Kit (NZ-51031, Enzo) according to the manufacturer's instructions. Briefly, 2.4×10^5^ cells were seeded in six-well plates overnight and then treated for 24 hours with 80 μg/ml PG or vehicle. The cells were then harvested and stained with fluorescent dyes to measure autophagic vacuoles, including preautophagosomes, autophagosomes, and autolysosomes, and monitor the autophagic flux. The florescence intensity and number were detected and measured using the NucleoCounter NC-3000 system (ChemoMetec, Allerod, Denmark).

### Lysosome formation

PG-induced lysosome formation was measured using the LYSO-ID Green detection kit (ENZ-51034, Enzo). The dye accumulates in acidic compartments, such as endosomes, lysosomes, and secretory vesicles. Briefly, 2.4×10^5^ cells were seeded in six-well plates overnight. After treatment with 80 μg/ml PG or vehicle for 48 h, cells were then harvested and stained with fluorescent dyes using the LYSO-ID Green detection kit and measured using the NucleoCounter NC-3000 system (ChemoMetec).

### Protein extraction and Western blot analysis

Cells were treated with PG or vehicle for 48 h. Proteins were analyzed by Western blotting as previously described [28]. Briefly, 20 μg of proteins were separated by sodium dodecyl sulfate-polyacrylamide gel electrophoresis (SDS-PAGE) and electrotransferred onto polyvinylidene difluoride membranes (GE Healthcare Piscataway, NJ, USA). Then incubated with ATG5, ATG12, Beclin-1, LC3, Bcl2, Bax, or c-PARP antibodies at 4°C overnight. Respective secondary antibody was subsequently probed and visualized using an enhanced chemiluminescence reagent (GE Healthcare Piscataway, NJ, USA) and detected using VersaDoc 5000 (Bio-Rad Laboratories, Hercules, CA, USA).

### Statistical analyses

The data are presented as the mean±standard deviation (SD) of at least three independent experiments. Significant differences were analyzed using Student’s *t*-test (two-tailed) to compare two groups, with p<0.05 considered significant.

## Results

### PG inhibits HCC cell growth

**Fig 1:**
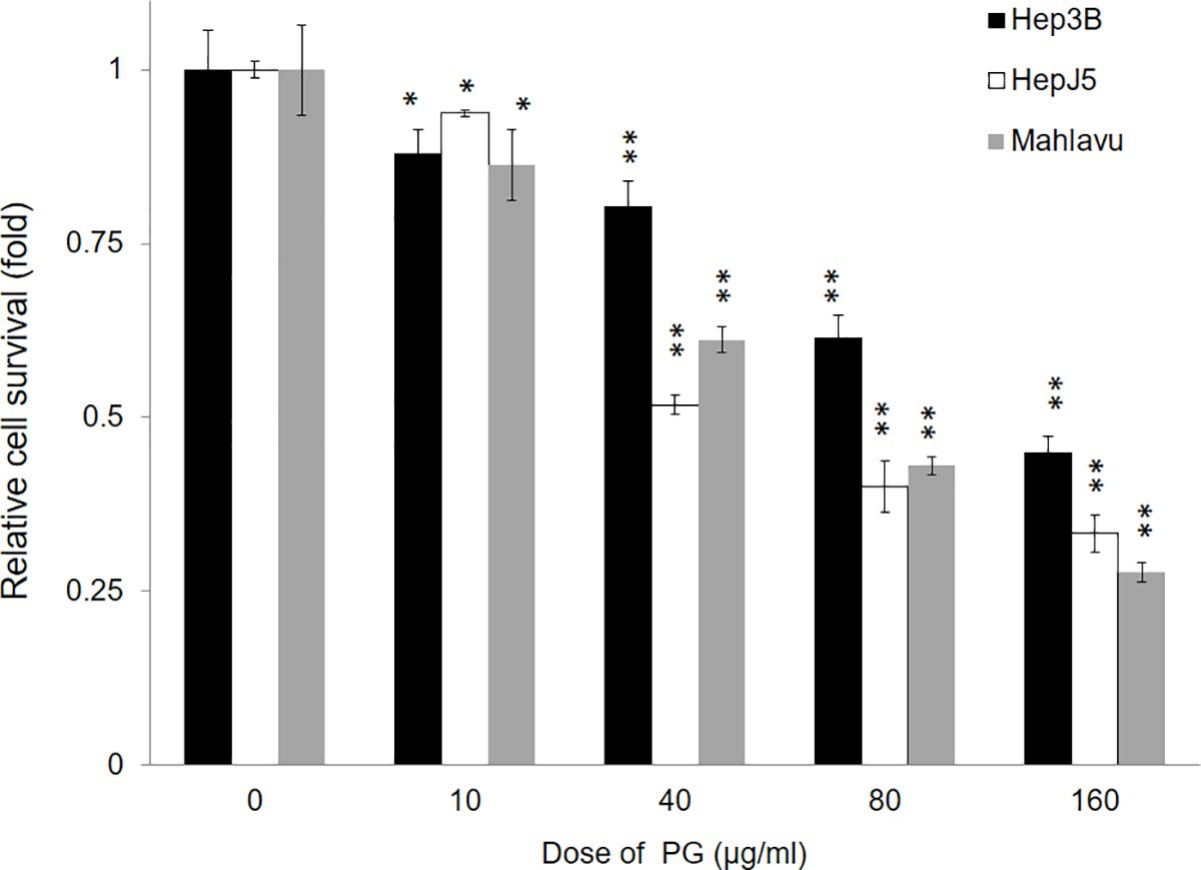
PG treatment decreases HCC cell survival. Hep3B, Mahlavu, and HepJ5 cells were treated with different concentrations of PG (0–160 μg/ml) for 48 h. The cell survival rate was determined using the SRB assay. The vehicle treatment was set at 100% survival. PG treatment reduced the cell survival rate in HCC cells in a dose-dependent manner. The data are presented as the mean±SD of three independent experiments in triplicate (** p<0.01, *p<0.05).

First, the cytotoxic effects of PG on HCC were investigated. Three HCC cells lines different in differentiated status were evaluated with the SRB assay, Hep3B, Mahlavu and HepJ5 cells. PG treatment markedly decreased the proliferation of HCC cell lines in a dose-dependent manner (Fig 1). The 50% maximum inhibitory concentration (IC_50_) values after 48 h of treatment with PG in the Hep3B, Mahlavu, and HepJ5 cell lines were 40–135 μg/ml, respectively (Table 1). Well differentiated Hep3B cell is more resistant to PG treatment than poor differentiated cell lines, Mahlavu and HepJ5 cells. Then we evaluated the most resistant Hep3B cell and most sensitive HepJ5 cells with xenotransplantation assay.

**Table 1:** The IC_50_ of propyl gallate (PG) on HCC cells.

Cells	IC _50_ for propyl gallate (μg/ml)
Hep3B	135.25
HepJ5	43.42
Mahlavu	64.74

### PG suppresses HCC cell proliferation in a zebrafish model

**Fig 2:**
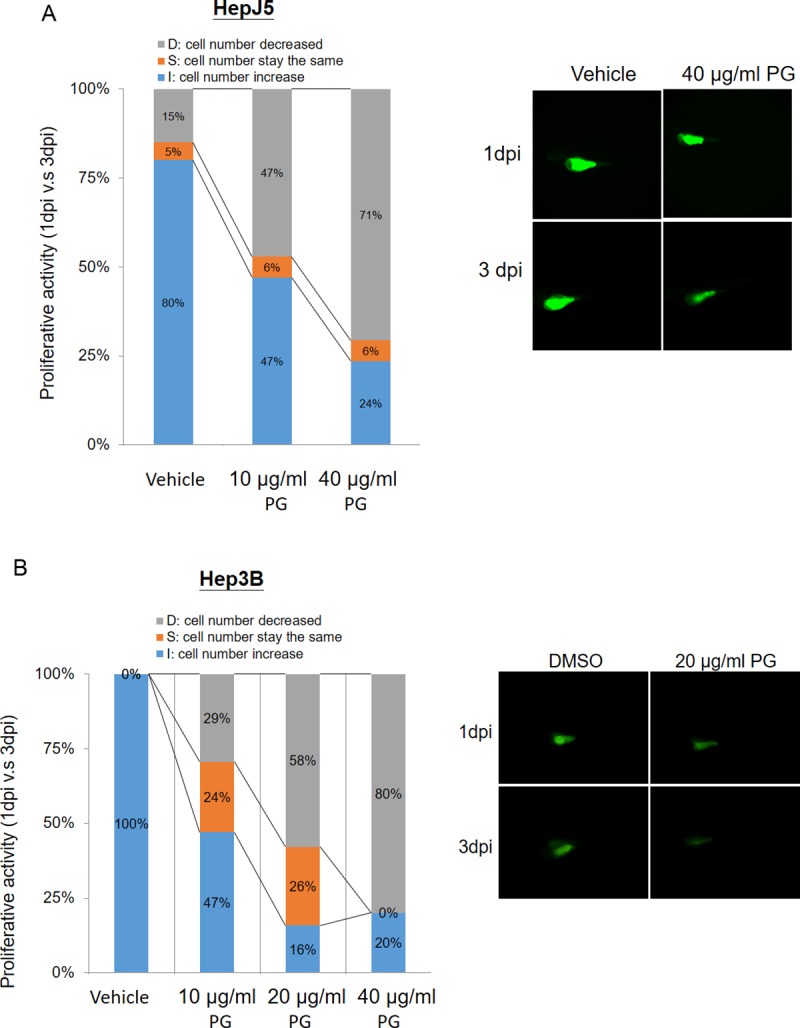
PG suppresses cell proliferation in a zebrafish model. The xenotransplantation assay was performed using zebrafish to determine the efficacy of PG treatment in HCC. Hep3B and HepJ5 cells were implanted into the embryo yolk and then exposed to different doses of PG (0–40 μg/ml). The proliferative activity in the HCC cell lines was compared by monitoring the fluorescence intensity on day 1 and day 3 post-injection (1 dpi and 3 dpi) of PG. (A) PG at concentrations of 10 μg/ml and 40 μg/ml reduced the cell number increase in the embryo population (from 80% vehicle to 47% and 24%, respectively) in HepJ5 cells. A decreased fluorescence intensity was shown after 3 days in HepJ5 cells with 40 μg/ml PG treatment. (B) In Hep3B cell lines, the cell number increase in the embryo population was decreased from 100% (vehicle) to 47%, 16% and 20% (10 μg/ml, 20 μg/ml, and 40 μg/ml, respectively). Treatment with 20 μg/ml PG dramatically decreased the fluorescence intensity in Hep3B cells compared with vehicle.

The role of PG treatment in HCC progression was further confirmed using a xenotransplantation assay performed with zebrafish. In the HepJ5 cells, the cell number increase in the embryo population was reduced from 80% (vehicle) to 47% and 24% (10 or 40 μg/ml PG, respectively) (Fig 2A). However, in Hep3B cell line, the cell number increase in the embryo population was reduced from 100% (vehicle) to 47%, 16% and 20% (10, 20, and 40 μg/ml, respectively) (Fig 2C). PG treatment also significantly decreased the fluorescence intensity in the two cell lines in a dpi-dependent manner compared with vehicle treatment (Fig 2B and 2D). These results indicate that PG inhibits the growth ability of HCC cell lines in a dose- and time-dependent manner.

### PG induces apoptosis in HCC cells by high-content screening

**Fig 3:**
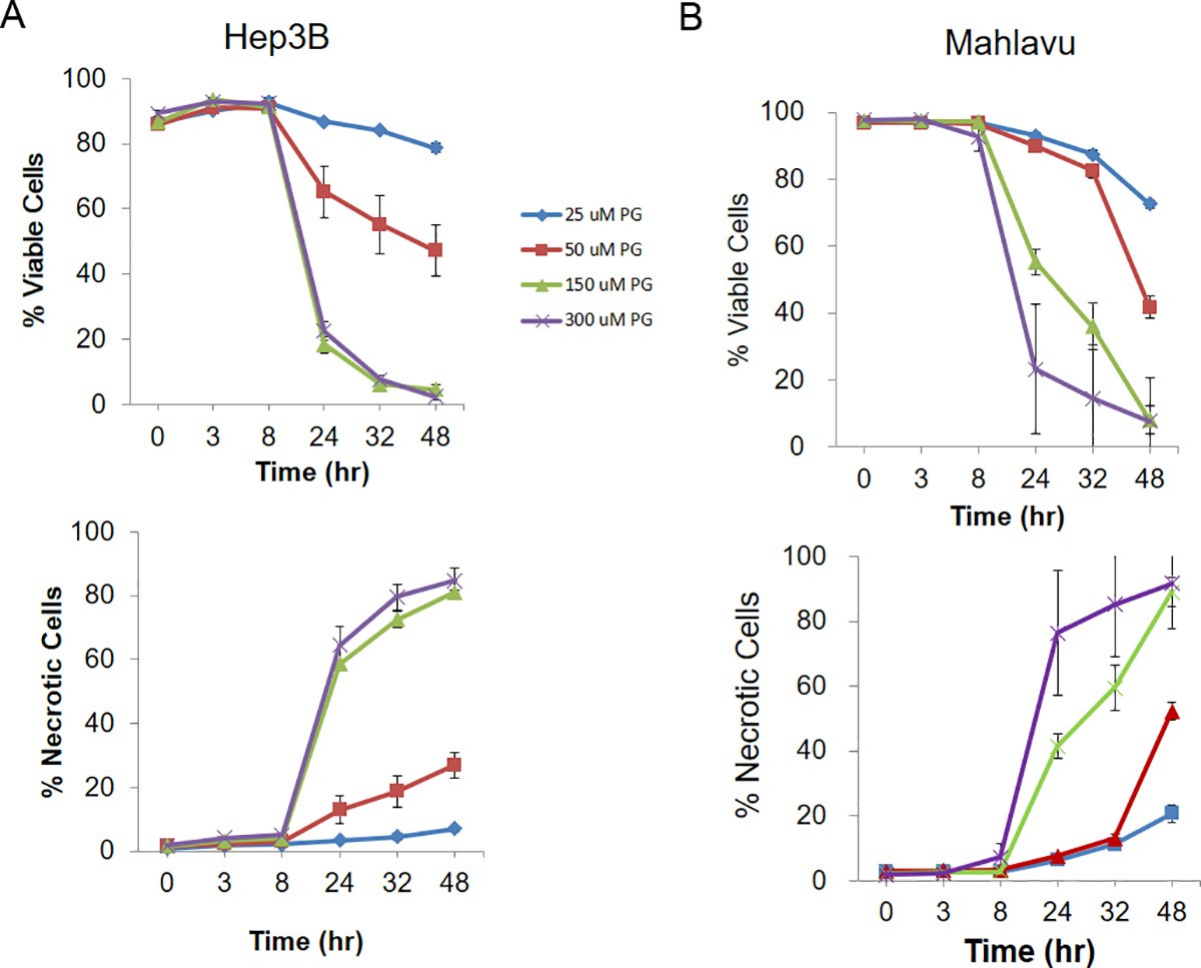
PG induces cell apoptosis in HCC cell lines. The percentage of viable and necrotic cells was measured by high-content analysis at different time intervals. (A) The percentage of viable cells was decreased after PG treatment in Hep3B cells. The necrotic signals were increased after PG treatment in a dose-dependent manner. (B) In Mahlavu cells, the number of viable cells was significantly decreased, whereas necrotic cells were increased after PG exposure. The data are presented as the mean±SD of three independent experiments in triplicate.

**Fig 4:**
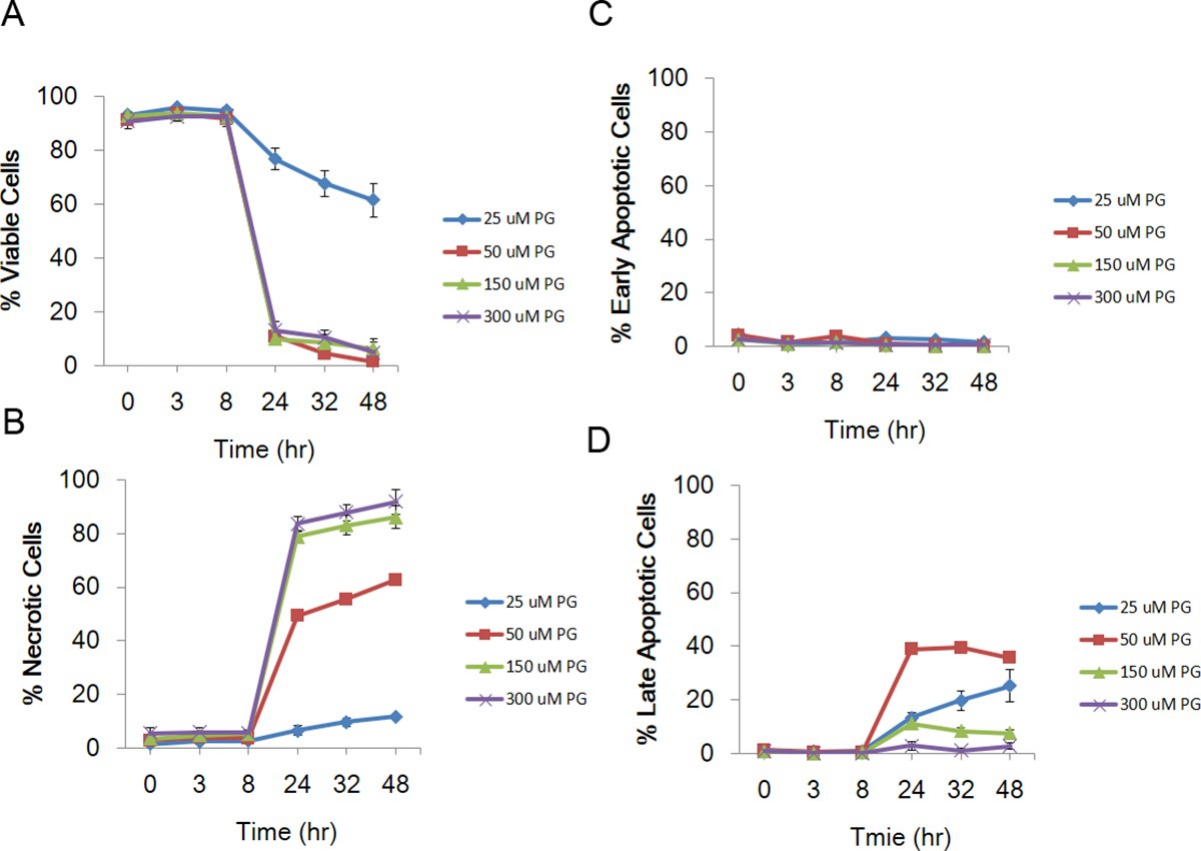
PG increased the number of necrotic cells in HepJ5 cells. The percentage of viable and necrotic cells was measured by high-content analysis at specific time intervals. (A) PG exposure caused a dramatic decrease in HepJ5 cell viability after 24 h. (B) PG caused an increase in the number of necrotic cells after a 24-h exposure. (C) Different PG doses did not induce early apoptosis. (D) The number of late apoptotic cells was increased after exposure to 50 μM PG in HepJ5 cells. The data are presented as the mean±SD of three independent experiments in triplicate.

To further understand the cell inhibition of PG, HCC cells were exposed to PG (0–300 μM), and the percentage of viable and necrotic cells was measured by high-content screening at different time intervals. As shown in Fig 3, high PG doses resulted in a decrease in cell viability and an increase in the number of necrotic cells in a time- and dose-dependent manner, mainly beginning after 8 h after PG exposure (Figs 3A, 3B, 4A and 4B). The percentage of early and late apoptotic cells was also measured in the HepJ5 cell line, which was shown to be the most sensitive of the three cell lines after exposure to the above high doses of PG and at the indicated time points. Although the different high doses of PG did not result in early apoptosis, late apoptosis was induced beginning 8 h after treatment with a 50 μM dose, resulting in more apoptotic cells (~40%) at 24 h, followed by the 25 μM dose. Notably, high doses of 150 or 300 μM did not result in a significant increase in apoptotic cells (Fig 4C and 4D).

### PG treatment causes cell apoptosis

**Fig 5:**
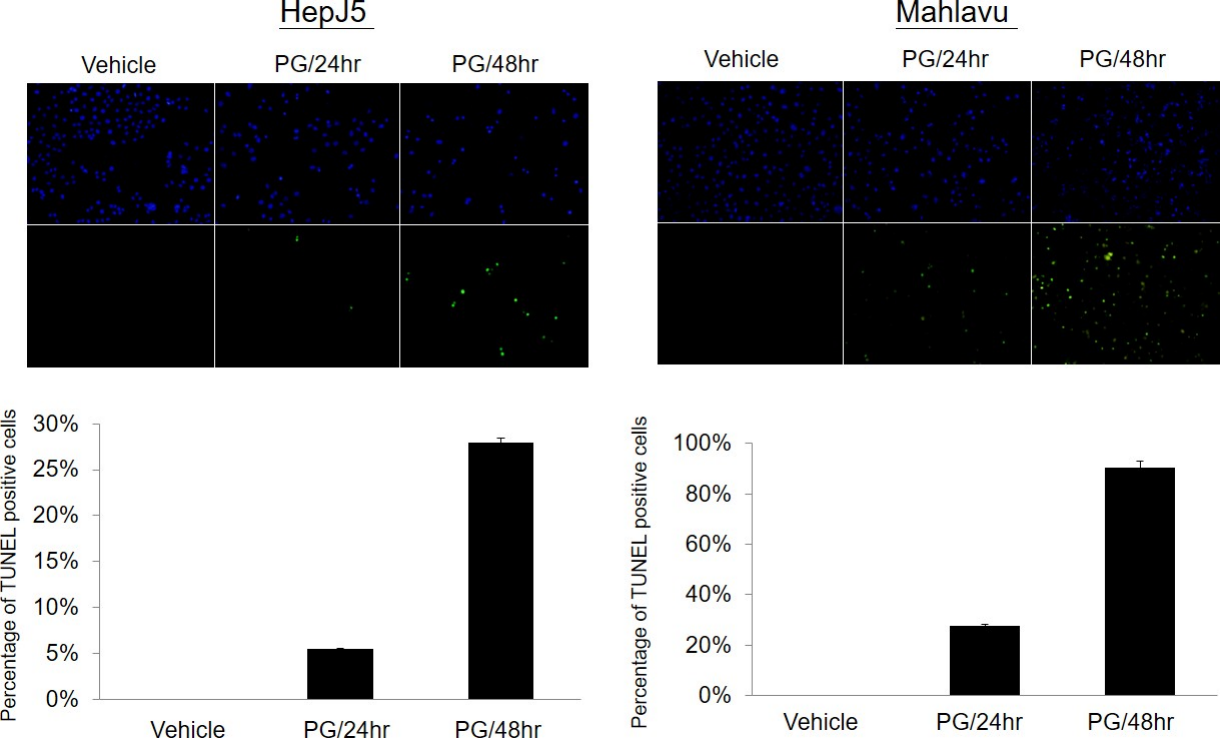
PG treatment induced cell apoptosis as monitored by a TUNEL assay. (A) Representative fluorescent figure of the apoptotic assay. Blue DAPI was used to stain nuclei and TdT tagged with a green fluorochrome was used to detect apoptotic DNA fragmentation. There were few cells with positive apoptotic signals in the vehicle control sample. However, exposure to 80 μg/ml PG for 24hr or 48hr induced dramatically more apoptotic signaling in a time-dependent manner.

To further confirm whether or not bromelain treatment can induce cell apoptosis, a TUNEL assay was applied. As shown in Fig 5, we found that there were few cells with positive signals in the vehicle control sample. However, after exposure to 80 μg/ml PG, numbers of cells with positive signals dramatically increased in a time-dependent manner. This indicated that PG inhibited cell proliferation through induction of apoptosis.

### PG enhances ROS and superoxide generation

**Fig 6:**
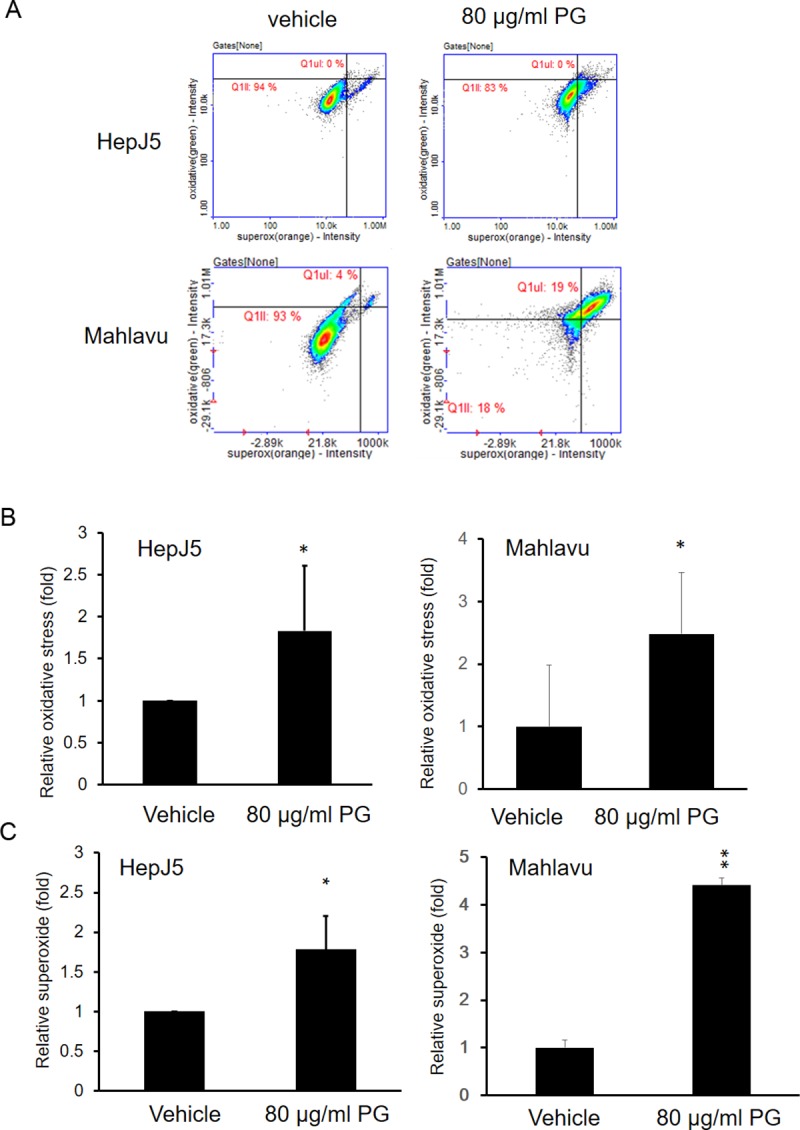
PG increases oxidative and superoxide levels in HepJ5 cells. HepJ5 cells were treated with 80 μg/ml PG for 24 h. The ROS and superoxide levels were detected using specific dyes. (A) The fluorescence intensity for oxidative or superoxide formation was detected by NC3000 in vehicle or PG-treated HepJ5 or Mahlavu cells. (B) The level of oxidative formation was increased in PG-treated HepJ5 and Mahlavu cells. (C) An significantly increased superoxide levels was found in PG-treated HepJ5 and Mahlavu cells compared with the vehicle-treated cells. The data are presented as the mean±SD of three independent experiments in triplicate (*p<0.05, **p<0.01).

ROS are involved in the induction of apoptosis in several systems. HepJ5 cells were used to determine the role of PG in ROS and superoxide production, which was assessed using the total ROS/Superoxide Detection Kit. As shown in Fig 6A, the intensities of oxidative or superoxide were increased after PG treatments in HepJ5 and Mahlavu cells. PG exposure significantly enhanced intracellular oxidative levels and superoxide generation in both HepJ5 and Mahlavu cells (Fig 6B and 6C). Those results indicate that PG exposure caused an increase of oxidative and superoxide production.

### PG treatment causes the induction of autophagy and lysosome formation

**Fig 7:**
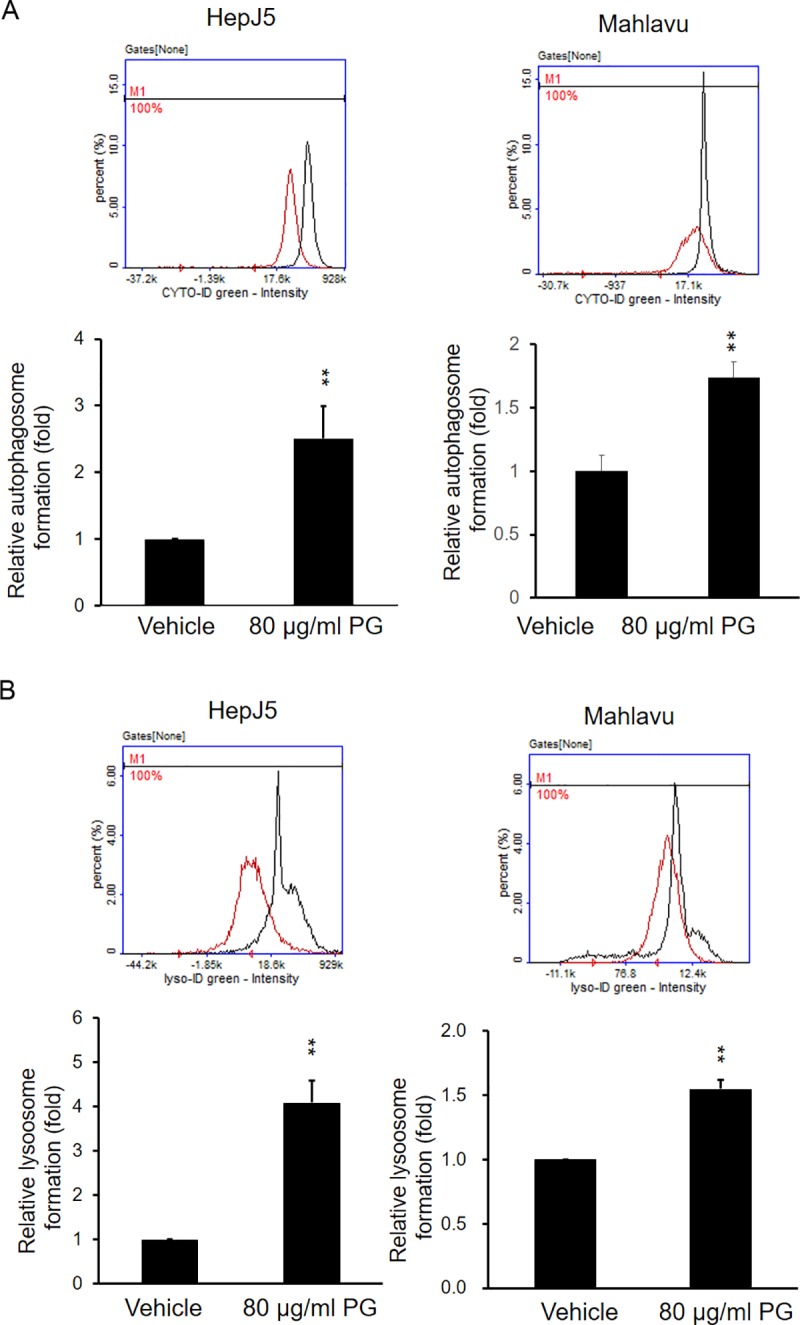
PG increases the formation of autophagosomes and lysosomes. HepJ5 cells were exposed to 80 μg/ml PG for specific times. Autophagosome and lysosomes were detected using specific dyes and detected by NC-3000. (A) The formation of autophagosomes was increased after PG treatment in HepJ5 and Mahlavu cells compared with the vehicle treatment. (B) The formation of autophagosomes and lysosomes was increased after PG treatment compared with the vehicle treatment in HepJ5 and Mahlavu cells. The data are presented as the mean±SD of three independent experiments in triplicate (**p<0.01).

The autophagy pathway plays an important role in cancer cell survival. We further investigated the autophagy status using the Enzo autophagy detection kit. As shown in Fig 7A, PG exposure for 24 h increased autophagy dramatically in both HepJ5 and Mahlavu cells. We further investigated lysosome formation. As shown in Fig 7B, lysosome formation was increased after PG exposure in HepJ5 and Mahlavu cells. Together, our results demonstrated that PG exposure may activate the autophagy pathway and lysosome formation.

### Pretreatment with AGH, an antioxidant, reverses PG-induced ROS formation

**Fig 8:**
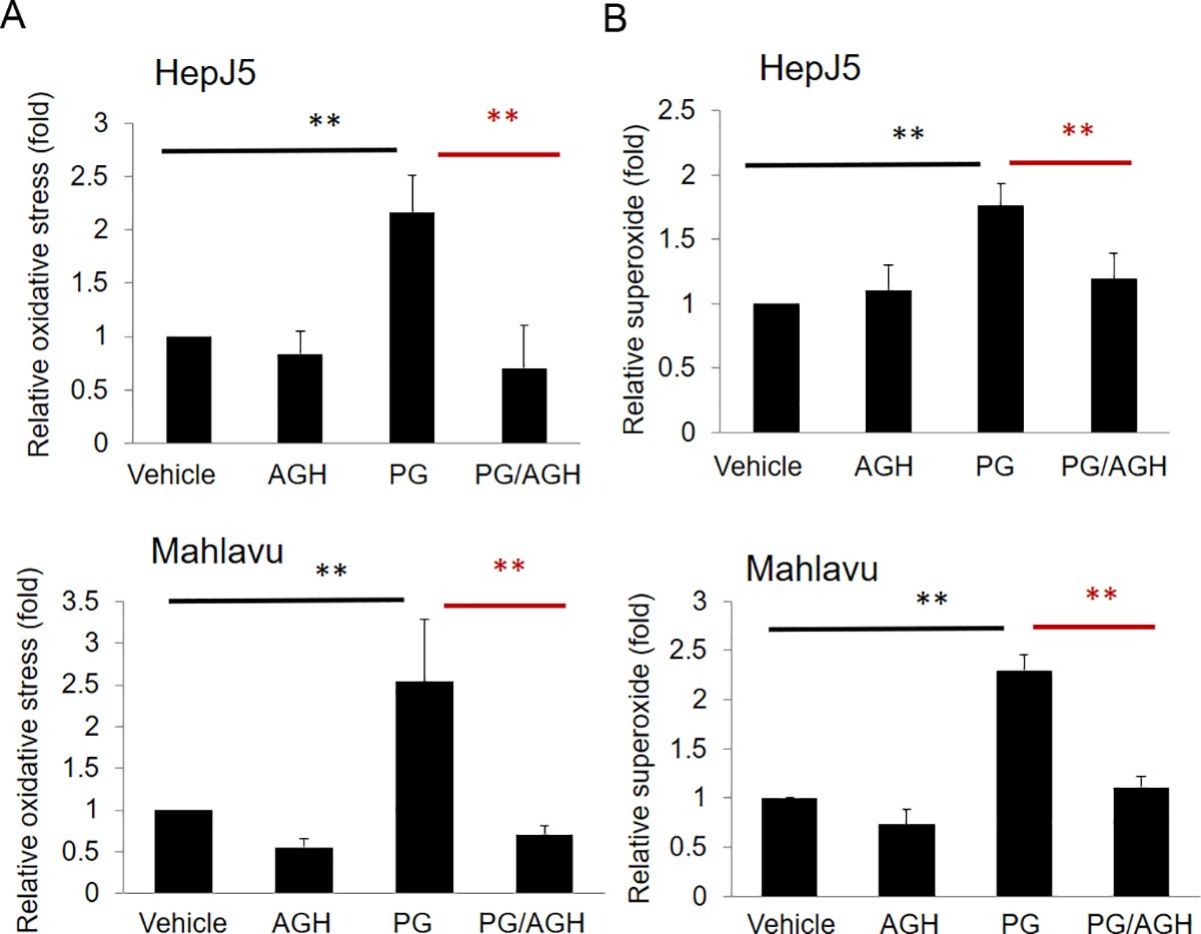
Pretreatment with AGH (antioxidant) abolishes the PG-induced ROS and superoxide levels. HepJ5 or Mahlavu cells were pretreated with AGH before exposure to PG. ROS and superoxide levels were detected using specific fluorescence dyes. (A) The ROS level increased significantly after PG treatment, and pretreatment with AGH in HepJ5 and Mahlavu cells abolished the PG-induced ROS production. (B) The superoxide level increased after exposure to PG, and pretreatment with AGH abolished the PG-induced superoxide production in HepJ5 and Mahlavu cells. The data are presented as the mean±SD of three independent experiments in triplicate (**p<0.01).

To further explore this phenomenon, aminoguanidine hemisulfate (AGH), a well-known antioxidant, and a diamine oxidase and nitric oxide synthase inhibitor [29], was used to determine whether it can reverse the effects of PG. As shown in Fig 8, PG exposure induces oxidative and superoxide generation. Interestingly, pretreatment with AGH prevented oxidative (Fig 8A) and superoxide (Fig 8B) formation after PG exposure. This result indicates that pretreatment with AGH significantly blocked ROS and superoxide levels in PG-treated HepJ5 and Mahlavu cells.

### PG affects the expression of proteins associated with apoptosis

**Fig 9:**
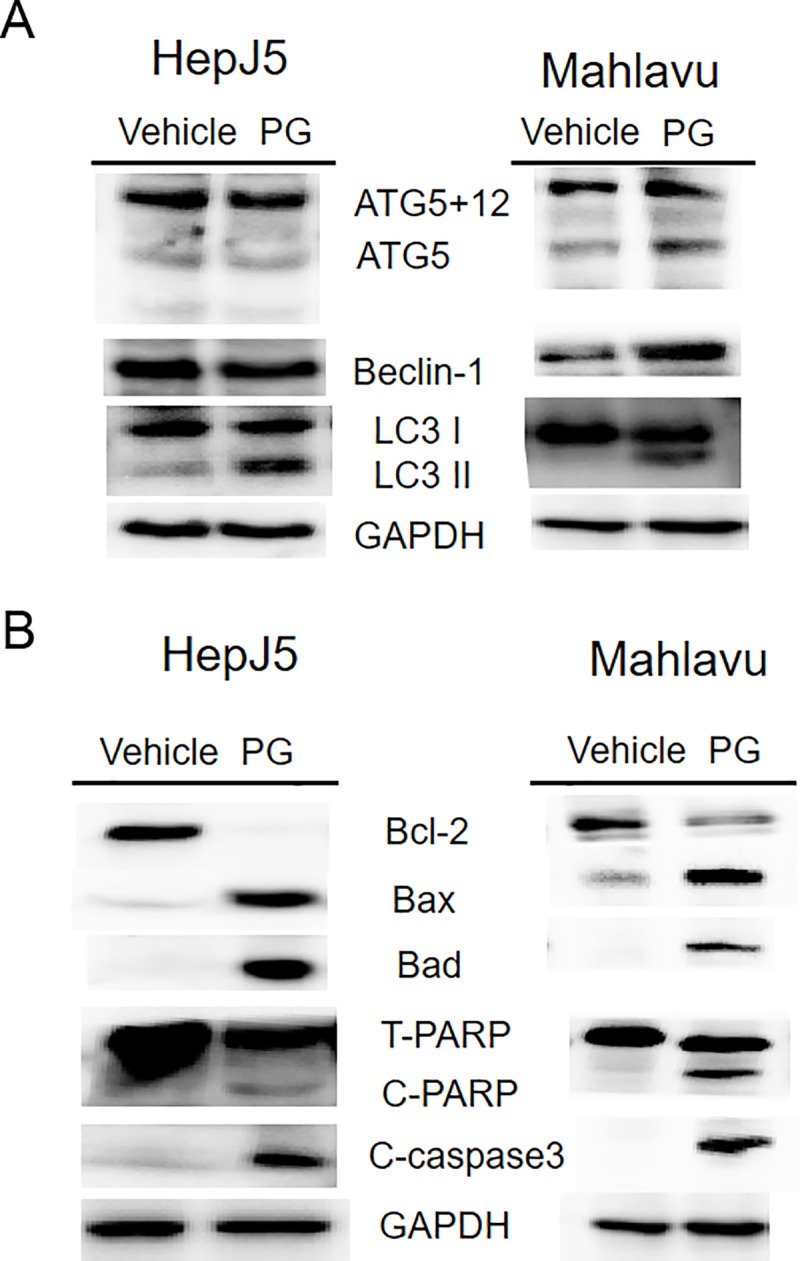
PG treatment causes changes in autophagy- and apoptosis-related proteins. HepJ5 or Mahlavu were treated with 80 ug/ml PG or vehicle for 24 h. **A**. The levels of autophagy related proteins (ATG5, ATG12, Beclin-1, LC3) were checked by western blotting. The amount of ATG5, ATG5+12, becklin-1 was similar between PG-treated and vehicle-treated sample. The ratio of LC3-II/LC3-I was high in PG treated sample. **B.** The cell apoptosis related proteins, Bcl-2, Bax, Bad, c-PARP, and c-caspase3 was checked. PG treated cells showed decreased Bcl-2 (anti-apoptotic), increased Bax and Bad protein (pro-apoptotic) and increased cleavage PARP (C-PARP) and cleavage caspase 3 (C-caspase3). All experiments were repeated at least three times independently.

Regarding the molecular mechanism, we evaluated the expression pattern of autophagy- and apoptosis-related proteins by Western blotting. As shown in Fig 9A, PG treatment did not result in a significant change in the expression of ATG5 or ATG12 in HepJ5 and Mahlavu cells. PG-treated Mahlavu cells caused an increase in beclin-1 level, but there is no influence in HepJ5 cells treated with PG. The rate of LC3-I to LC3-II conversion was increased after PG treatment, indicating the induction of autophagy. In addition, PG treatment caused an increase in the pro-apoptotic proteins Bax, Bad, cleaved PARP and cleaved caspase-3 but a decrease in the anti-apoptotic Bcl-2 (Fig 9B). Those results are consistent with our previous findings.

## Discussion

Owing to the vaccination, screening, and therapeutic developments, the survival rate of localized HCC has significantly improved. Disappointingly, the long-term prognosis of advanced HCC remained unchanged over the past 30 years [30]. It is urgent to identify novel effectively treatments through different anticancer mechanisms. In the presented study, we examined the anticancer effects of PG in HCC cells. We observed that PG induced a dose-dependent inhibition of the proliferation of HCC cell lines and that ROS-mediated apoptosis and autophagy contributes to PG-induced cell death in HCC cells.

Apoptosis is the most comprehensive form of programmed cell death that can be divided into intrinsic and extrinsic apoptosis pathways. Caspases play a cardinal role in both intrinsic and extrinsic apoptosis [31]. The Bcl-2 family proteins consist of both anti- and pro-apoptotic members. The intrinsic apoptosis is mediated by the Bcl-2 family, which includes pro-apoptotic proteins, such as Bax, Bak, and Bad, as well as anti-apoptotic proteins, such as Bcl-2 and Bcl-XL [32]. Here, upregulated levels of cleaved caspase-3 and PARP were observed in HCC cells, which were accompanied by a significant decrease in Bcl-2 and an increase in Bax and Bad, indicating that PG could trigger intrinsic apoptosis to induce cell death in HCC cells. We observed the early and late stages of apoptosis, and even necrosis, in HCC cells treated with PG. Our results confirmed that the apoptosis of HCC cells induced by PG may mediated via the mitochondria-specific pathway.

Autophagy is processed when cells need to produce intracellular nutrients and energy, and cells consume their unfolded proteins and cytoplasmic organelles to maintain cellular homeostasis [33]. Autophagy can be classified as macroautophagy, microautophagy, and chaperone-mediated autophagy (CMA) based on the process of delivery of cargo into the lysosome. The term “autophagy” is usually used to direct to macroautophagy. In cancer, autophagy can be harmless, tumor-suppressive, or tumor-promoting in different circumstances[34, 35]. This process is tightly mediated by a set of regulating distinct proteins. Autophagy has been reported for its dual role in tumor progression. Recent oncological reports have related autophagy to clinical chemotherapy and radiotherapy failure [36]. Cancer cells induce autophagy under stressful environments as a solution of cell survival; however, sustained autophagy finally leads a type II programmed cell death. The autophagic process is encoded by more than 30 autophagy related genes (ATG) [37]. Autophagy-related 5 (ATG5) is a key protein involved in autophagic vesicles that is activated by ATG7 and forms a complex with ATG12 and ATG16. This complex is necessary for the conversion of LC3-II (LC3-phosphatidylethanolamine conjugate) to LC3-I [38]. Recent studies have shown that autophagy flux is not greatly linked to the change in ATG expression at the mRNA or protein levels [39]. Here, we found that PG treatment showed no significant effects on Beclin-1, ATG5 and ATG12 expression. During autophagy progression, LC-3 is cleaved to LC3-II, which localizes and aggregates onto the membranes of autophagosomes, demonstrating the autophagy condition. We observed that PG-induced autophagy in HCC cells was characterized by increased vacuoles of the autophagosome and lysosome and the conversion of the autophagy marker LC3-I to LC3-II. The LC3-I and LC3-II expression levels serve as good indicators of autophagic monitoring.

PG is a generally recognized as safe (GRAS) antioxidant in foods and cosmetic products at a maximum concentration of 0.1%. It is currently used as an antioxidant to protect food from peroxides induced rancidity [40]. In addition, PG may increase antifungal drug efficacy against filamentous fungi [41]. Previous studies have shown the apoptosis-inducing characteristics of PG, which can delay the growth of hepatic stellate cells to inhibit liver fibrosis [17, 19, 42, 43]. Hepatitis B/C virus infection and/or alcohol-induced liver inflammation play a critical role in multi-step processes of liver injury and carcinogenesis [44]. HBV-, HCV- and alcohol- triggered oxidative stress and induce liver damage[45]. It could be of therapeutic value in protecting the liver from injury, inflammation, and carcinogenesis [46–51]. There are several possible agents for preventing stress related liver pathogenesis, such as curcumin [52, 53], silymarin [54], green tea [55], and vitamins C or E [56]. The antioxidative abilities of PG can be altered into prooxidative properties in the presence of certain ions, such as Cu^2+^ [16, 57]. Thus, PG functions as cytotoxic agent rather than cytoprotective antioxidant. ROS levels, including peroxides and superoxides, are increased in PG-treated HCC cells as we have demonstrated. ROS are chemically reactive molecules that may arise during environmental stress. ROS play a significant role in oxidative stress and are generated as by-products of mitochondrial metabolism [58]. Increasing of ROS leads to loss of the mitochondrial membrane potential (MMP) and cytochrome C releasing from damaged mitochondria [59]. The results of the present study indicated that ROS generation is related to apoptosis and autophagy in PG-treated HCC cells.

Many anticancer drugs and natural compounds have been reported to have antitumor effects via ROS-induced cell apoptosis. Cancer cells adapted strict environment, but are particularly sensitive to prolonged and increased ROS. ROS production in mitochondria induces programmed cell death, functioning in an upstream apoptotic pathway. Our studies showed that PG inhibits HCC cell proliferation through the regulation of apoptosis-related signaling pathways. On the other hand, excess mitochondrial ROS has been demonstrated to modulate the autophagy process [60, 61]. In response to a cellular stress, autophagy is a cellular bulk degradation system to maintain organelle metabolism and survival. In cancer cells, the functional relationship between apoptosis and autophagy is complex. ROS could modulate the fate of cancer cells through modulating various signaling pathways, including cell cycle arrest, apoptosis and autophagy. Thus, further studies are necessary to reveal the underlying mechanisms in the future.

DNA damage and mitochondrial dysfunction elevated ROS in cells response to multiple stimuli, including and are implicated in the modulation of apoptosis and autophagy. ROS levels modulate cell death through the activation of various signaling pathways, including AKT/mTOR or Erk/Akt/NFkB pathways [62, 63]. Previous study showed that PG inhibits migration in malignant glioma cells through ROS modulation and NF-*κ*B Pathway inhibition [64]. PG-induced cell death, via apoptosis or autophagy, is dependent on ROS production. PG-triggered ROS and superoxide were markedly reversed by AGH, a well-known antioxidant. Furthermore, apoptosis and autophagy induced by PG in HCC cells in vitro were additionally recapitulated in vivo using Hep3B and HepJ5 cells in the experimental zebrafish xenograft model.

Our results demonstrate that PG inhibits human HCC cells via apoptosis and autophagy. Multiple hypothetical pathways have been discussed previously, and Chen, C.H., et al. showed in leukemia cells [17] the activation of MAPKs and caspases, the upregulation of p53, Bax, Fas, and Fas-L expression and GSH depletion. Interestingly, PG-induced GSH depletion and cell death in leukemia cells did not occur by increasing ROS levels in leukemia cells. PG-induced inhibition of Nrf-2 nuclear translocation, sequenced by c-GCS downregulation, may finally result in GSH depletion in PG-treated leukemia cells. In our result, it’s interesting that well differentiated Hep3B cell is more resistant to PG treatment than poor differentiated cell lines, HepJ5 and Mahlavu cells. Many studies showed that different phenotypes in cancer cell lines exhibit different characters in morphological shapes and Epithelial-Mesenchymal Transition (EMT) status [65]. Different responses to hypoxia, starvation, therapeutic stress, or heat shock were also noted between different cell lines even in the same cancer, such as CRC or HCC [28, 66]. It finally determines the fate of cancer cells to PG treatment may be related to the distinct ROS status, EMT profiles, hypoxia inducible factors (HIF), or heat shock protein levels[65]. PG may play a different role at the ROS level, and the cellular response depends on different PG concentrations, treatment duration, or cell type. Therefore, further study is indicated for screening a promising selective target in patients with HCC undergoing responsive PG treatment in individualized medicine.

In conclusion, the presented study shows that PG with various anticancer effects as a therapeutic choice for HCC. PG treatment induces growth inhibition of HCC cells both in vitro and in vivo. PG-induced HCC cells death, through the mitochondria-related apoptosis, involves Bcl-2, Bax, Bad, caspase and cleaved PARP. PG induces intracellular ROS and superoxide, and the upregulation of LC3-II is essential for the induction of PG-induced autophagy in HCC cells. Our results suggest that PG may be a potential clinical agent to treat HCC.
